# Underlying SUSY in a generalized Jaynes–Cummings model

**DOI:** 10.1038/s41598-021-95259-1

**Published:** 2021-08-13

**Authors:** F. H. Maldonado-Villamizar, C. A. González-Gutiérrez, L. Villanueva-Vergara, B. M. Rodríguez-Lara

**Affiliations:** 1 CONACYT-Instituto Nacional de Astrofísica, Óptica y Electrónica, Calle Luis Enrique Erro No. 1, Sta. Ma. Tonantzintla, Pue., 72840 Puebla, Mexico; 2 Instituto de Nanociencia y Materiales de Aragón (INMA) and Departamento de Física de la Materia Condensada, CSIC-Universidad de Zaragoza, Zaragoza, 50009 Zaragoza, Spain; 3 Instituto Nacional de Astrofísica, Óptica y Electrónica, Calle Luis Enrique Erro No. 1, Sta. Ma. Tonantzintla, Pue., 72840 Puebla, Mexico; 4 Tecnologico de Monterrey, Escuela de Ingeniería y Ciencias, Ave. Eugenio Garza Sada 2501, 64849 Monterrey, NL Mexico

**Keywords:** Nonlinear optics, Quantum optics, Atom optics, Applied mathematics

## Abstract

We present a general qubit-boson interaction Hamiltonian that describes the Jaynes–Cummings model and its extensions as a single Hamiltonian class. Our model includes non-linear processes for both the free qubit and boson field as well as non-linear, multi-boson excitation exchange between them. It shows an underlying algebra with supersymmetric quantum mechanics features allowing an operator based diagonalization that simplifies the calculations of observables. As a practical example, we show the evolution of the population inversion and the boson quadratures for an initial state consisting of the qubit in the ground state interacting with a coherent field for a selection of cases covering the standard Jaynes–Cummings model and some of its extensions including Stark shift, Kerr-like, intensity dependent coupling, multi-boson exchange and algebraic deformations.

## Introduction

The standard model of particle physics classifies all elemental physical objects into fermions and bosons^1,2^. Under this unified theory for three of the four fundamental forces, particles acquire masses through the Higgs mechanism via scalar bosons^3^. The renormalization of the scalar masses of these bosons shows a hierarchy problem: they diverge^4,5^. The theoretical idea of supersymmetry (SUSY), for each boson there must exist a SUSY fermion partner and vice-versa, presents a way to circumvent these divergences as the contributions from SUSY partners should have opposite signs^6,7^.

It is possible to construct an oversimplification of these theories in the form of SUSY quantum mechanics (SUSY-QM) of Witten index two if we consider just one boson and a fermion^8^. For example, we describe them by their annihilation (creation) operators, ${\hat{f}}$ and ${\hat{a}}$ (${\hat{f}}^{\dagger }$ and ${\hat{a}}^{\dagger }$), in that order, fulfilling the canonical relations $\{ {\hat{f}}, {\hat{f}}^{\dagger } \} = [ {\hat{a}}, {\hat{a}}^{\dagger }] = 1$, and construct two SUSY operators that exchange a fermion (boson) by a boson (fermion),

1 $$\begin{aligned} {\hat{Q}}= {\hat{f}} {\hat{a}}^{\dagger } \quad ({\hat{Q}}^{\dagger } = {\hat{f}}^{\dagger } {\hat{a}}), \end{aligned}$$

by annihilating the former (latter) and creating the latter (former). These non-Hermitian exchange operators are nilpotent,

2 $$\begin{aligned} {\hat{Q}}^{2} = {\hat{Q}}^{\dagger 2} = 0, \end{aligned}$$

as once we exchange the SUSY partners we are not able to perform the same operation again. However, we may create an operator that is a sequence of both processes,

3 $$\begin{aligned} {\hat{H}} = \left\{ {\hat{Q}}^{\dagger } , {\hat{Q}} \right\} = {\hat{Q}}^{\dagger } {\hat{Q}} + {\hat{Q}} {\hat{Q}}^{\dagger }, \end{aligned}$$

and leaves the original configuration unchanged. This operator receives the name of SUSY Hamiltonian. The first (second) term in the rightmost side takes a fermion (boson) into a boson (fermion) and back into a fermion (boson) and, thus, is known as the fermionic (bosonic) sector, ${\hat{H}}_{F} = {\hat{Q}}^{\dagger } {\hat{Q}}$ (${\hat{H}}_{B} = {\hat{Q}} {\hat{Q}}^{\dagger }$), of the SUSY Hamiltonian ${\hat{H}}$. As a consequence, it is a constant of motion,

4 $$\begin{aligned} \left[ {\hat{H}} , {\hat{Q}} \right] = \left[ {\hat{H}} , {\hat{Q}}^{\dagger } \right] = 0, \end{aligned}$$

of the SUSY-QM model with Witten index two and provides the so-called intertwining relations, ${\hat{Q}} {\hat{H}}_{F} = {\hat{H}}_{B} {\hat{Q}}$ and ${\hat{H}}_{F} {\hat{Q}}^{\dagger } = {\hat{Q}}^{\dagger } {\hat{H}}_{B}$, between the two sectors. The exchange operators are non-Hermitian but we may use them to construct Hermitian operators,

5 $$\begin{aligned} {\hat{Q}}_{X} = {\hat{Q}}^{\dagger } + {\hat{Q}} \quad \text {and} \quad {\hat{Q}}_{Y} = -i \left( {\hat{Q}}^{\dagger } - {\hat{Q}} \right) , \end{aligned}$$

known as SUSY charges whose squares yield the SUSY Hamiltonian,

6 $$\begin{aligned} {\hat{H}} = {\hat{Q}}_{X}^{2} = {\hat{Q}}_{Y}^{2} = \frac{1}{2} \left( {\hat{Q}}_{X}^{2} + {\hat{Q}}_{Y}^{2}\right) . \end{aligned}$$

The fact that the SUSY Hamiltonian may be expressed as the square of Hermitian operators suggest that the spectrum should be double degenerate and that the minimum energy state should be zero. When the minimum energy state is not zero, the spectrum of both fermionic and bosonic sectors are identical and we say that SUSY is broken^5,9^.

Quantum technologies provide multiple experimental platforms where a single pseudo-fermion and boson degrees of freedom interact; for example, two-internal levels of a neutral atom interacting with a single mode of the quantum electromagnetic field^10^, those of a trapped ion interacting with a quantum center of mass vibration mode^11^, a superconducting Josephson junction interacting with the quantum mode of a strip-line resonator^12^, or a quantum dot interacting with a two-dimensional photonic resonator^13^. In these realizations, we may write the SUSY exchange operators and Hamiltonian,

7 $$\begin{aligned} {\hat{Q}}= {\hat{\sigma }}_{-} {\hat{a}}^{\dagger }, \quad {\hat{Q}}^{\dagger } = {\hat{\sigma }}_{+} {\hat{a}}, \quad {\hat{H}} = {\hat{\sigma }}_{+} {\hat{\sigma }}_{-} {\hat{a}} {\hat{a}}^{\dagger } + {\hat{\sigma }}_{-} {\hat{\sigma }}_{+} {\hat{a}}^{\dagger } {\hat{a}}, \end{aligned}$$

in terms of up (down) Pauli operators for the pseudo-fermion ${\hat{\sigma }}_{+}$ (${\hat{\sigma }}_{-}$), hereafter called a qubit, and creation (annihilation) operators for the boson mode ${\hat{a}}^{\dagger }$ (${\hat{a}}$). The fermionic sector, ${\hat{Q}}^{\dagger } {\hat{Q}} = {\hat{\sigma }}_{+} {\hat{\sigma }}_{-} {\hat{a}} {\hat{a}}^{\dagger }$, has eigenstates $\vert f; n \rangle = \vert e, n \rangle$ with eigenvalues $E_{f,n} = n + 1$ and those corresponding to the bosonic sector, ${\hat{Q}} {\hat{Q}}^{\dagger } = {\hat{\sigma }}_{-} {\hat{\sigma }}_{+} {\hat{a}}^{\dagger } {\hat{a}}$, are $\vert b; n \rangle = \vert g, n \rangle$ with eigenvalues $E_{b,n} = n$, where the notation $\vert g \rangle$ ($\vert e \rangle$) refers to the ground (excited) state of the qubit and, for a Fock state of the boson field, we write $\vert n \rangle$ with $n=0,1,2,3, \ldots$ The minimum energy state of the SUSY Hamiltonian is unique, belongs to the bosonic sector, and has zero value. Thus, SUSY is unbroken. The rest of the spectrum has fermionic-bosonic SUSY partners for each subsequent energy level $E_{f,n} = E_{b,n+1}$. This SUSY Hamiltonian shows dispersive interaction between the qubit and the boson field^14,15^, with no actual excitation exchange between them and it may be difficult to implement in the laboratory. For example, an original proposal uses an interaction free qubit and boson field with the anti-Jaynes–Cummings model in the strong coupling regime^16^; this regime has just been recently made available for trapped-ion^12^ and superconducting circuit^17^ quantum electrodynamics experiments. However, the SUSY charges,

8 $$\begin{aligned} {\hat{Q}}_{X} = {\hat{\sigma }}_{+} {\hat{a}} + {\hat{\sigma }}_{-} {\hat{a}}^{\dagger } \quad \text {and} \quad {\hat{Q}}_{Y} = -i \left( {\hat{\sigma }}_{+} {\hat{a}} - {\hat{\sigma }}_{-} {\hat{a}}^{\dagger } \right) \end{aligned}$$

are the heart of the quantum optics workhorse, the Jaynes–Cummings model,^18^

9 $$\begin{aligned} {\hat{H}} = \omega {\hat{a}}^{\dagger } {\hat{a}} + \frac{1}{2} \omega _{0} {\hat{\sigma }}_{z} + g \left( {\hat{\sigma }}_{+} {\hat{a}} + {\hat{\sigma }}_{-} {\hat{a}}^{\dagger } \right) , \end{aligned}$$

describing the interaction of a qubit, with energy gap proportional to the frequency $\omega _{0}$ and the Pauli operators, and a boson field with frequency $\omega$ and described by the annihilation and creation operators. The interaction strength *g* has values that depend on the particular experimental realization. SUSY-QM already helped understanding the Jaynes–Cummings model providing a class of ladder operators that lead to interesting coherent states using standard diagonalization techniques^19^.

We are interested in finding a more general qubit-boson interaction model that allows us to describe the Jaynes–Cummings model and its extensions. In particular, we want to keep an underlying algebra with SUSY characteristics that helps us provide an operator based diagonalization of the model that may simplify the calculations of observables of the system. In the following, we propose a generalization of the Jaynes–Cummings model that includes non-linear processes for both the free qubit and boson field as well as non-linear, multi-boson excitation exchange between them, “Generalized Jaynes–Cummings model”. Our model helps realize that the original Jaynes–Cummings and most of its proposed extensions in the literature belong to a single Hamiltonian class. In “Graded Lie algebra”, we show that our model presents an underlying algebra with SUSY characteristics. Then, in “Diagonalization”, we transform the Hamiltonian to diagonal form using our proposed algebra and we find the eigenstates and the time evolution of observables of interest in “Eigenstates and time evolution”. For the sake of providing a practical example, we use our results to visualize the dynamics of specific realizations of our Hamiltonian class that have been discussed through the years in the literature, “Particular cases”, starting from the Jaynes–Cummings model. Finally, we close with our conclusion in “Conclusion”.

## Generalized Jaynes–Cummings model

The introduction of the Jaynes–Cummings model^18^, to describe the interaction of a two-level system with a boson field under the rotating wave approximation (RWA), opened the door to more complicated models from both the theoretical and experimental perspectives. We focus on a generalized Jaynes–Cummings model,

10 $$\begin{aligned} {\hat{H}} = \omega {\hat{n}} +\frac{1}{2} \omega _{0} {\hat{\sigma }}_{z}+ {\hat{\sigma }}_{z} F({\hat{n}}) + G({\hat{n}}) + g \left[ {\hat{\sigma }}_{+} f({\hat{n}}){\hat{a}}^{k} + {\hat{\sigma }}_{-} {\hat{a}}^{\dagger k}f({\hat{n}}) \right] , \end{aligned}$$

that accounts for families of reported models and more. Here, the frequency $\omega _{0}$ provides the qubit energy gap and the up (down) and population inversion operators, ${\hat{\sigma }}_{+}$ (${\hat{\sigma }}_{-}$) and ${\hat{\sigma }}_{z}$, provide its dynamics. The boson field frequency is $\omega$ with creation (anihilation) and boson excitation number operators, ${\hat{a}}^{\dagger }$ (${\hat{a}}$) and ${\hat{n}} \equiv {\hat{a}}^{\dagger } {\hat{a}}$, in that order. The RWA approximation, $\vert k\omega -\omega _{0}\vert \ll k\omega +\omega _{0}$ requires $\vert g\vert \ll \omega _{0}$. The first two terms in the right hand side of Eq. (10) are the energy of the free qubit and the boson field. The third term implies nonlinear shifting of the spectrum as a function of the boson excitation number; it includes the Stark effect. The fourth term is a collection of nonlinear effects in the boson field; it includes the Kerr effect. The fifth term is the nonlinear, multi-boson interaction between the qubit and the boson field under the rotating wave approximation.

Our model covers but is not limited to a cohort of examples from the literature. The obvious one is the Jaynes–Cummings model^18^, describing the interaction of a qubit with a boson field in the RWA and, as discussed in the introduction, relates to standard supersymmetric quantum mechanics^19^. One of the first extensions of the Jaynes–Cummings model used an intensity dependent coupling and multiboson exchange interaction^20,21^. Soon after, a slight modification included nonlinear effects such as Kerr-like terms and two-boson exchange^22–25^. In these works, photon statistics and time evolution of physical observables were presented. The addition of the Stark shift, an interesting effect describing the qubit energy gap dependence on the intensity of the field, came later^26,27^. Nonlinear extensions for the occupation number were proposed as a generalization to the Kerr effect^28^. Then, trapped ions were proposed to realize nonlinear multiboson exchange interaction^29^. An algebraic generalizaton was proposed to study coherent states for an anharmonic perturbation to the Jaynes–Cummings model. Some of us studied a, slightly complicated in hindsight, generalization^30–35^ that reduces to our general scheme in the following section.

## Graded Lie algebra

Let us focus on just the interaction part of our generalized Jaynes–Cummings Hamiltonian and recast it into the form,

11 $$\begin{aligned} {\hat{H}}_{I} = g \left( {\hat{{\mathscr {Q}}}}^{\dagger } + {\hat{{\mathscr {Q}}}} \right) , \end{aligned}$$

where we define the nilpotent exchange operators,

12 $$\begin{aligned} {\hat{{\mathscr {Q}}}}^{\dagger } = {\hat{\sigma }}_{+} f({\hat{n}}) {\hat{a}}^{k}, \quad \text {and} \quad {\hat{{\mathscr {Q}}}} = {\hat{\sigma }}_{-} {\hat{a}}^{\dagger k} f({\hat{n}}), \end{aligned}$$

such that ${\hat{{\mathscr {Q}}}}^{\dagger 2} = {\mathscr {Q}}^{2} = 0$. These provide the SUSY Hamiltonian,

13 $$\begin{aligned} \begin{aligned} \hat{{\mathscr {H}}} & = \left\{ \hat{{\mathscr {Q}}}^{\dagger } , \hat{{\mathscr {Q}}} \right\} , \\ &={\hat{\sigma }}_{+}{\hat{\sigma }}_{-} {\hat{a}}^{k}{\hat{a}}^{\dagger k}f^{2}({\hat{n}}) + {\hat{\sigma }}_{-}{\hat{\sigma }}_{+} {\hat{a}}^{\dagger k}{\hat{a}}^{k}f^{2}({\hat{n}}-k), \end{aligned} \end{aligned}$$

that commutes with the exchange operators $\left[ \hat{{\mathscr {H}}}, \hat{{\mathscr {Q}}}^{\dagger } \right] = \left[ \hat{{\mathscr {H}}}, \hat{{\mathscr {Q}}} \right] = 0$, and whose diagonal elements are the isospectral fermionic and bosonic sectors,

14 $$\begin{aligned} \begin{aligned} \hat{{\mathscr {H}}}_{F} \vert e, n \rangle &=\hat{{\mathscr {Q}}}^{\dagger } \hat{{\mathscr {Q}}} \vert e, n \rangle = f^{2}(n)\dfrac{(n+k)!}{n!} \vert e, n \rangle , \\ \hat{{\mathscr {H}}}_{B} \vert g, n \rangle &=\hat{{\mathscr {Q}}} \hat{{\mathscr {Q}}}^{\dagger } \vert g, n \rangle = f^{2}(n-k)\dfrac{n!}{(n-k)!} \vert g, n \rangle , \end{aligned} \end{aligned}$$

connected by the intertwining relations,

15 $$\begin{aligned} \hat{{\mathscr {Q}}} \hat{{\mathscr {H}}}_{F} = \hat{{\mathscr {H}}}_{B} \hat{{\mathscr {Q}}}, \qquad \text {and} \qquad \hat{{\mathscr {H}}}_{F} \hat{{\mathscr {Q}}}^{\dagger } = \hat{{\mathscr {Q}}}^{\dagger } \hat{{\mathscr {H}}}_{B}. \end{aligned}$$

It is possible to define two charge operators,

16 $$\begin{aligned} \hat{{\mathscr {Q}}}_{X} = \hat{{\mathscr {Q}}}^{\dagger } + \hat{{\mathscr {Q}}} \qquad \text {and} \qquad \hat{{\mathscr {Q}}}_{Y} =- i \left( \hat{{\mathscr {Q}}}^{\dagger } - \hat{{\mathscr {Q}}} \right) , \end{aligned}$$

that are the square root of the Hamiltonian $\hat{{\mathscr {Q}}}_{X}^{2} = \hat{{\mathscr {Q}}}_{Y}^{2} = {\mathscr {H}}$. Thus, the interaction part of our generalized Jaynes–Cummings model is proportional to the square root of the Hamiltonian $\hat{{\mathscr {H}}}$ with an underlying SUSY algebra^36–38^.

Now, let us recast our complete Hamiltonian,

17 $$\begin{aligned} {\hat{H}} = \omega \left( \hat{{\mathscr {N}}} - \hat{{\mathscr {B}}} \right) + \frac{\omega _{0}}{k} \hat{{\mathscr {B}}} + F(\hat{{\mathscr {N}}} - \hat{ {\mathscr {B}}}) \frac{2\hat{{\mathscr {B}}}}{k} + G \left( \hat{{\mathscr {N}}} - \hat{ {\mathscr {B}}} \right) + g \hat{{\mathscr {Q}}_{X}}, \end{aligned}$$

in terms of our algebra. Here, we define the total excitation number,

18 $$\begin{aligned} \hat{{\mathscr {N}}} = {\hat{n}} + \hat{{\mathscr {B}}}, \end{aligned}$$

in terms of the boson number operator ${\hat{n}}$ and the scaled Pauli z-matrix,

19 $$\begin{aligned} \hat{{\mathscr {B}}}=\frac{k}{2} {\hat{\sigma }}_{z}. \end{aligned}$$

We assume that the nonlinear boson functions are continuous and differentiable, such that $F(x)= \sum _{j} F_{j}x^{j}$ and $G(x)= \sum _{j} G_{j}x^{j}$ with the shorthand notation $f_{j} = d^{j} f(x) / dx^{j} \vert _{x=0}$. Under these conditions, both the SUSY Hamiltonian and the total excitation number commute with all other operators involved in our model,

20 $$\begin{aligned} \left[ \hat{{\mathscr {O}}}_{j} , \hat{{\mathscr {H}}} \right] = \left[ \hat{{\mathscr {O}}}_{j} , \hat{{\mathscr {N}}} \right] = 0, \end{aligned}$$

where the place holder operator $\hat{{\mathscr {O}}}_{j}$ stands for elements of the set $\hat{{\mathscr {O}}} = \left\{ \hat{{\mathscr {Q}}}, \hat{{\mathscr {Q}}}^{\dagger }, \hat{{\mathscr {H}}}, \hat{{\mathscr {N}}}, \hat{{\mathscr {B}}}\right\}$. The commutation relations between the charges and the scaled Pauli-z operator,

21 $$\begin{aligned} \left[ \hat{{\mathscr {B}}}, \hat{{\mathscr {Q}}}^{\dagger } \right] = k \hat{{\mathscr {Q}}}^{\dagger }, \quad \left[ \hat{{\mathscr {B}}}, \hat{{\mathscr {Q}}} \right] = - k \hat{{\mathscr {Q}}}, \quad \left[ \hat{{\mathscr {Q}}}^{\dagger }, \hat{{\mathscr {Q}}}\right] = \frac{2}{k} \hat{{\mathscr {H}}} \hat{{\mathscr {B}}}, \end{aligned}$$

are reminiscent of a deformed *su*(2) algebra. These relations will come handy in the diagonalization of our model.

## Diagonalization

It is possible to use the properties of the scaled Pauli-z operator to recast our generalized Jaynes–Cummings Hamiltonian in the form,

22 $$\begin{aligned} {\hat{H}} = \omega \hat{{\mathscr {N}}} + {\mathscr {F}} (\hat{{\mathscr {N}}}) + \left[ \frac{\omega _{0}}{k} - \omega + {\mathscr {G}}(\hat{{\mathscr {N}}}) \right] \hat{{\mathscr {B}}} + g \hat{{\mathscr {Q}}}_{X}, \end{aligned}$$

where the auxiliary functions in terms fo the total excitation number relate to the nonlinear boson number functions in the following manner,

23 $$\begin{aligned} \begin{aligned} {\mathscr {F}} (\hat{{\mathscr {N}}}) =&\sum _{j=0}^{\infty }\sum _{s=0}^{j}\left( {\begin{array}{c}j\\ 2s\end{array}}\right) \left( \dfrac{k}{2}\right) ^{2s}G_{j}{\hat{\mathscr {N}}}^{j-2s} -\sum _{j=0}^{\infty }\sum _{s=0}^{j}\left( {\begin{array}{c}j\\ 2s+1\end{array}}\right) \left( \frac{k}{2}\right) ^{2s-2}F_{j}{\hat{\mathscr{N}}}^{j-2s-1},\\ {\mathscr {G}}(\hat{{\mathscr {N}}}) =&-\sum _{j=0}^{\infty }\sum _{s=0}^{j}\left( {\begin{array}{c}j\\ 2s+1\end{array}}\right) \left( \frac{k}{2}\right) ^{2s}G_{j}{\hat{\mathscr{N}}}^{j-2s-1} + \sum _{j=0}^{\infty }\sum _{s=0}^{j} \left( {\begin{array}{c}j\\ 2s\end{array}}\right) \left( \dfrac{k}{2}\right) ^{2s-2}F_{j}{\hat{\mathscr{N}}}^{j-2s}. \end{aligned} \end{aligned}$$

Here, we used the fact that $\hat{{\mathscr {B}}}^{2 j} = ( k /2)^{2j}$ and $\hat{{\mathscr {B}}}^{2 j + 1} = (k/2)^{2j} \hat{{\mathscr {B}}}$. As the total number excitation is a conserved quantity of the model, the first two terms in the right hand side of Eq. (22) only introduce a phase factor. We move into a rotating frame defined by these terms,

24 $$\begin{aligned} \vert \psi \rangle = e^{-i \left[ \omega \hat{{\mathscr {N}}} + {\mathscr {F}} (\hat{{\mathscr {N}}}) \right] t} \vert \phi \rangle , \end{aligned}$$

such that we obtain an effective Hamiltonian,

25 $$\begin{aligned} {\hat{H}}_{\phi } = \left[ \frac{\omega _{0}}{k} - \omega + {\mathscr {G}}(\hat{{\mathscr {N}}}) \right] \hat{{\mathscr {B}}} + g \hat{{\mathscr {Q}}}_{X}, \end{aligned}$$

where the factor accompanying the scaled Pauli-z operator $\hat{{\mathscr {B}}}$ commutes with all other operators.

Now, we draw inspiration from standard diagonalization techniques for the Jaynes–Cummings model^19^, and propose a change of reference frame,

26 $$\begin{aligned} {\hat{D}}({\hat{\theta }}) = e^{ i {\hat{\theta }}(\hat{{\mathscr {N}}},\hat{{\mathscr {H}}}) \hat{{\mathscr {Q}}}_{Y}}, \end{aligned}$$

in terms of an Hermitian operator parameter ${\hat{\theta }}(\hat{{\mathscr {N}}},\hat{{\mathscr {H}}})$ that depends on the total excitation number $\hat{{\mathscr {N}}}$ and the SUSY Hamiltonian $\hat{{\mathscr {H}}}$. Thus, it will commute with all other elements of the algebra $\left[ {\hat{\theta }}, \hat{{\mathscr {O}}}_{j}\right] =0$, and we may use it as a parameter to diagonalize our generalized Jaynes–Cummings Hamiltonian in the new reference frame. It is cumbersome but straightforward to get a useful form,

27 $$\begin{aligned} {\hat{D}}({\hat{\beta }}) = e^{ i \frac{{\hat{\beta }}}{2} \hat{{\mathscr {H}}}^{-1/2} \hat{{\mathscr {Q}}}_{Y}}, \end{aligned}$$

that yields an effective diagonal Hamiltonian,

28 $$\begin{aligned} {\hat{H}}_{D} &={\hat{D}}^{\dagger }({\hat{\beta }}) {\hat{H}} {\hat{D}}({\hat{\beta }}), \nonumber \\ &= \left\{ \left[ \frac{\omega _{0}}{k} - \omega + {\mathscr {G}}(\hat{{\mathscr {N}}}) \right] \cos {\hat{\beta }} + \frac{2g}{k}\hat{{\mathscr {H}}}^{1/2} \sin {\hat{\beta }} \right\} \hat{{\mathscr {B}}}, \end{aligned}$$

for a displacement parameter operator fulfilling,

29 $$\begin{aligned} \tan {\hat{\beta }} = \frac{2g}{k}\hat{{\mathscr {H}}}^{1/2} \left[ \frac{\omega _{0}}{k} - \omega + {\mathscr {G}}(\hat{{\mathscr {N}}}) \right] ^{-1}. \end{aligned}$$

All involved terms are diagonal in the qubit and Fock basis and we used the expressions,

30 $$\begin{aligned} \begin{aligned} {\hat{D}}^{\dagger }({\hat{\beta }}) \hat{{\mathscr {B}}} {\hat{D}}({\hat{\beta }}) =&~ \hat{{\mathscr {B}}} \cos {\hat{\beta }} - \dfrac{k}{2}\hat{{\mathscr {H}}}^{-1/2}\hat{{\mathscr {Q}}_{X}} \sin {\hat{\beta }},\\ {\hat{D}}^{\dagger }({\hat{\beta }}) \hat{{\mathscr {Q}}_{X}} {\hat{D}}({\hat{\beta }}) =&~ \hat{{\mathscr {Q}}_{X}} \cos {\hat{\beta }} + 2\hat{{\mathscr {H}}}^{1/2} \hat{{\mathscr {B}}} \sin {\hat{\beta }}. \end{aligned} \end{aligned}$$

We want to stress that our unitary displacement operator,

31 $$\begin{aligned} {\hat{D}}({\hat{\beta }})=\cos ({\hat{\beta }})+\frac{1}{2}{\mathscr {H}}^{-1/2}\hat{{\mathscr {Q}}_{Y}}\sin ({\hat{\beta }}), \end{aligned}$$

reduces to that obtained for the Jaynes–Cummings model following standard diagonalization techniques^19^ upon substitution of adequate parameters and up to an overall phase provided by Eq. (24).

## Eigenstates and time evolution

In the original frame, it is possible to calculate the eigenstates of our model in terms of the manifold $\left\{ \vert {e, n} \rangle , \vert {g, n + k} \rangle \right\}$ with total excitation number ${\mathscr {N}} = \langle \hat{{\mathscr {N}}} \rangle = n + k/2$,

32 $$\begin{aligned} \begin{aligned} \vert + , {\mathscr {N}} \rangle =&~ {\hat{D}}({\hat{\beta }}) \vert e, n \rangle , \\ =&~ \cos \left( \dfrac{\beta ({\mathscr {N}})}{2}\right) \vert e,n \rangle + \sin \left( \dfrac{\beta ({\mathscr {N}})}{2}\right) \vert g, n+k\rangle , \\ \vert - , {\mathscr {N}} \rangle =&~ {\hat{D}}({\hat{\beta }}) \vert g, n + k \rangle ,\\ =&~ - \sin \left( \dfrac{\beta ({\mathscr {N}})}{2}\right) \vert e,n \rangle + \cos \left( \dfrac{\beta ({\mathscr {N}})}{2}\right) \vert g, n+k\rangle , \end{aligned} \end{aligned}$$

up to a common phase $\phi ({\mathscr {N}}) = \omega \left( n + k/2 \right) + F( n + k/2)$ and the relation

33 $$\begin{aligned} \tan \beta ({\mathscr {N}}) =&~ \frac{2g}{k}\sqrt{\frac{\left( {\mathscr {N}}+\frac{k}{2}\right) !}{\left( {\mathscr {N}}-\frac{k}{2}\right) !}}f\left( {\mathscr {N}}-\frac{k}{2}\right) \left[ \frac{\omega _{0}}{k} - \omega + {\mathscr {G}}\left( {\mathscr {N}}\right) \right] ^{-1}. \end{aligned}$$

The corresponding eigenvalues,

34 $$\begin{aligned} E_{\pm }({\mathscr {N}},k) =\pm \Omega \left( {\mathscr {N}}\right) , \end{aligned}$$

involve a generalized Rabi frequency,

35 $$\begin{aligned} \Omega ^{2}({\mathscr {N}})=\left[ \dfrac{\omega _{0}}{k}-\omega +{\mathscr {G}}\left( {\mathscr {N}}\right) \right] ^{2}+\frac{4g^{2}}{k^{2}}\frac{\left( {\mathscr {N}}+\frac{k}{2}\right) !}{\left( {\mathscr {N}}-\frac{k}{2}\right) !}~f^{2}\left( {\mathscr {N}}-\frac{k}{2}\right) . \end{aligned}$$

These results yield a time evolution in the diagonal frame,

36 $$\begin{aligned} {\hat{U}}(t) = e^{-i \left\{ \left[ \frac{\omega _{0}}{k} - \omega + {\mathscr {G}}(\hat{{\mathscr {N}}}) \right] \cos {\hat{\beta }} + \frac{2g}{k}\hat{{\mathscr {H}}}^{1/2} \sin {\hat{\beta }} \right\} \hat{{\mathscr {B}}} t}, \end{aligned}$$

that helps us calculate the evolution of the Pauli-z operator

37 $$\begin{aligned} \langle {\hat{\sigma }}_{z}(t) \rangle&=\langle \psi (0)\vert D({\hat{\beta }})\left[ {\hat{\sigma }}_{z} \cos {\hat{\beta }} + \hat{{\mathscr {H}}}^{-1/2}\left( \hat{{\mathscr {Q}}}^{\dagger }e^{ik{\hat{H}}_{D}\hat{{\mathscr {B}}}^{-1}t} + \hat{{\mathscr {Q}}}e^{-ik{\hat{H}}_{D}\hat{{\mathscr {B}}}^{-1}t} \right) \sin {\hat{\beta }}\right] D^{\dagger }({\hat{\beta }}) \vert \psi (0) \rangle . \end{aligned}$$

For example, assuming an initial state with the qubit in the ground state and the boson field in a Fock state,

38 $$\begin{aligned} \vert \psi (0) \rangle = \vert g, n\rangle , \end{aligned}$$

it is straightforward to calculate,

39 $$\begin{aligned} \langle {\hat{\sigma }}_{z}\rangle _{{\mathscr {N}}}&= \cos ^{2}\beta ({\mathscr {N}})+\sin ^{2}\beta ({\mathscr {N}})\cos \left[ \Omega ({\mathscr {N}})t\right] . \end{aligned}$$

The other observable, the boson field excitation number, is trivial,

40 $$\begin{aligned} \langle {\hat{n}} \rangle = \langle \hat{{\mathscr {N}}} \rangle - \frac{k}{2} \langle {\hat{\sigma }}_{z} \rangle . \end{aligned}$$

We use these expressions to compare several models included in our generalized Hamiltonian involving an initial state with the qubit in the ground state and the boson in a coherent state,

41 $$\begin{aligned} \vert \psi (0)\rangle =\vert g, \alpha \rangle =\sum _{j=0}^{\infty }\frac{e^{-\frac{\vert \alpha \vert ^{2}}{2}}}{\sqrt{j!}}\alpha ^{j}\vert g, j\rangle . \end{aligned}$$

The evolution of the Pauli-z operator is,

42 $$\begin{aligned} \langle g,\alpha \vert {\hat{\sigma }}_{z}\vert g, \alpha \rangle =\sum _{j=0}^{\infty }\frac{e^{-\vert \alpha \vert ^{2}}}{j!}\vert \alpha \vert ^{2j}\langle {\hat{\sigma }}_{z}(t) \rangle _{{\mathscr {N}}}, \end{aligned}$$

where $\langle \hat{\sigma _{z}}\rangle _{{\mathscr {N}}}$ is that in Eq. (39) and ${\mathscr {N}}=j-\frac{k}{2}$. This series does not converge to a closed expression but it is possible to approximate it for each particular case using known methods^39^. In general, the evolution of the population inversion for an initial coherent state, Eq. (42), involves the sum of single but fixed Rabi frequencies terms, Eq. (35).

## Particular cases

Our contribution focus on identifying that there exists a Hamiltonian class with an underlying graded Lie algebra that provides us with a unitary transformation to diagonalize our model. However, we want to show how simple it is to use our results to analyze the dynamics of some particular cases of our model^40^. In all cases, Fig. Xa shows the time evolution of the population inversion for an initial state involving a coherent state. Figure Xb and Fig. X(c) show the evolution of the boson quadratures,

43 $$\begin{aligned} {\hat{x}} = \frac{1}{2} \left( {\hat{a}}^{\dagger } + {\hat{a}} \right) ~~\text {and}~~{\hat{y}} = \frac{i}{2} \left( {\hat{a}}^{\dagger } - {\hat{a}} \right) , \end{aligned}$$

in polar plot form where the real mean value of the quadratures is the radial coordinate and time is the polar coordinate.

### Jaynes–Cummings model

**Figure 1:**
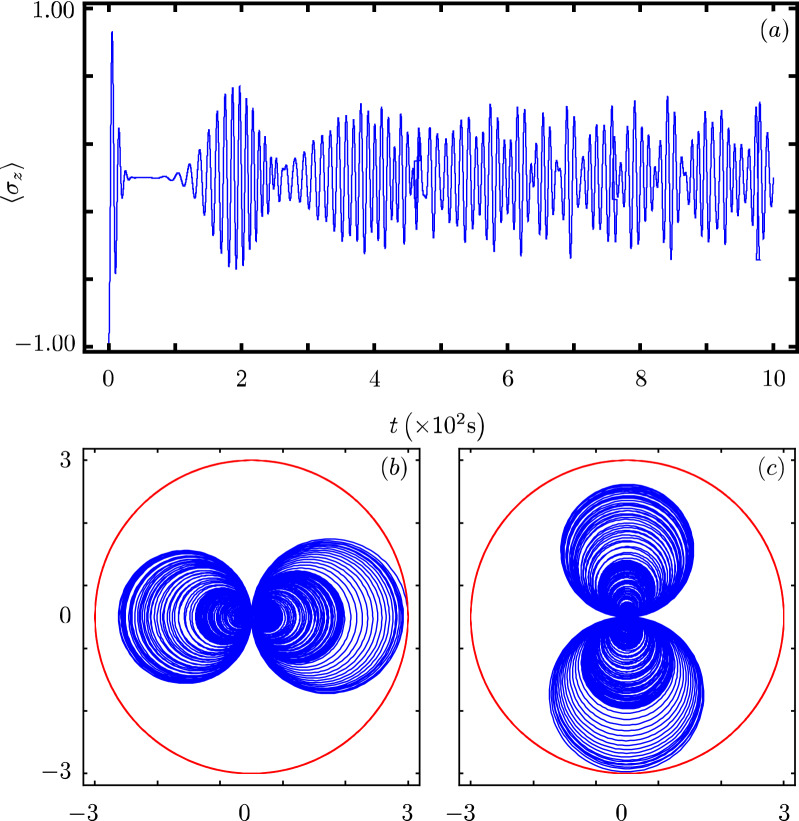
Time evolution of the (**a**) population inversion, (**b**) *x*-quadrature and (**c**) *y*-quadrature for the JC model, that is, our model with parameters $\omega = \omega _{0}$, $G({\hat{n}})=0$, $F({\hat{n}})=0$, $f({\hat{n}})=1$, $g=0.1 \omega _{0}$ and $k=1$, for an initial state with the qubit in the ground state and the boson in a coherent state with $\alpha =3$.

Figure 1 shows one of the most theoretically studied and experimentally tested models in quantum optics^11,17,18,41^. The Jaynes–Cummings (JC) model,

44 $$\begin{aligned} {\hat{H}}_{1}=\omega {\hat{n}}+\frac{\omega _0}{2}\sigma _{z}+g({\hat{\sigma }}_{+}{\hat{a}}+{\hat{\sigma }}_{-}{\hat{a}}^{\dagger }), \end{aligned}$$

allows the identification $G({\hat{n}})=0$, $F({\hat{n}})=0$, $f({\hat{n}})=1$ and $k=1$. For an initial coherent state, its population inversion shows so-called collapse and revival, Fig. 1a. Its quadratures show how the boson state is squeezed as time evolves, Fig. 1b,c.

### JC model with intensity-dependent multi-boson coupling

**Figure 2:**
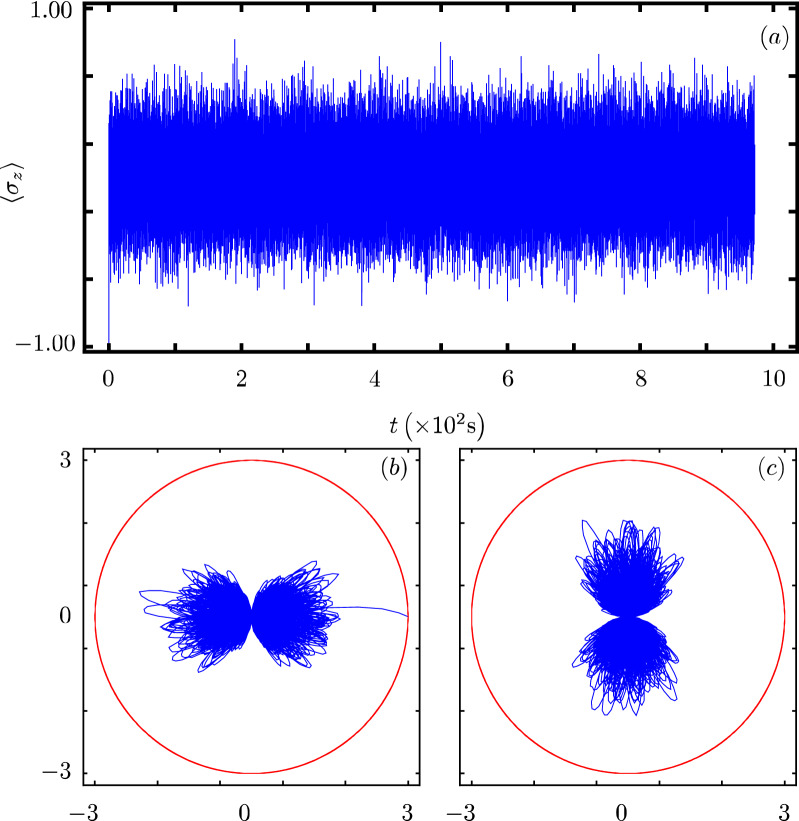
Same as Fig. 1 with $f({\hat{n}})=\sqrt{{\hat{n}}}$ and $k=2$.

One of the first extensions of the standard JC model included multiboson exchange and intensity dependent coupling $f({\hat{n}})={\hat{n}}^{1/2}$^42,43^,

45 $$\begin{aligned} {\hat{H}}_{2}=\omega {\hat{n}}+\frac{\omega _0}{2}{\hat{\sigma }}_z +g({\hat{\sigma }}_+{\hat{a}}^m\sqrt{{\hat{n}}}+{\hat{\sigma }}_-\sqrt{{\hat{n}}}{\hat{a}}^{\dagger m}), \end{aligned}$$

leading to $G({\hat{n}})=0$, $F({\hat{n}})=0$, and $f({\hat{n}})=\sqrt{{\hat{n}}}$. The evolution of its populations inversion is well known for initial Fock states^20^. For an initial coherent state, its population inversion oscillates with a fast frequency around a value of zero, Fig. 2a. Its quadratures show that the boson state is squeezed in a faster manner and explores a more localized portion of optical phase space than in the JC case, Fig. 2b,c.

### JC model with two-photon interaction and Stark shift

This model essentially implement an additional term describing how the field intensity effects the qubit energy gap^22,23,44^,

46 $$\begin{aligned} {\hat{H}}_{3}=\omega {\hat{n}}+{\hat{n}}\frac{\beta _2+\beta _{1}}{2}+\frac{\omega _0}{2}{\hat{\sigma }}_{z}+{\hat{n}}\frac{\beta _2-\beta _{1}}{2}{\hat{\sigma }}_{z} +g({\hat{\sigma }}_+{\hat{a}}^2+{\hat{\sigma }}_-{\hat{a}}^{\dagger 2}). \end{aligned}$$

**Figure 3:**
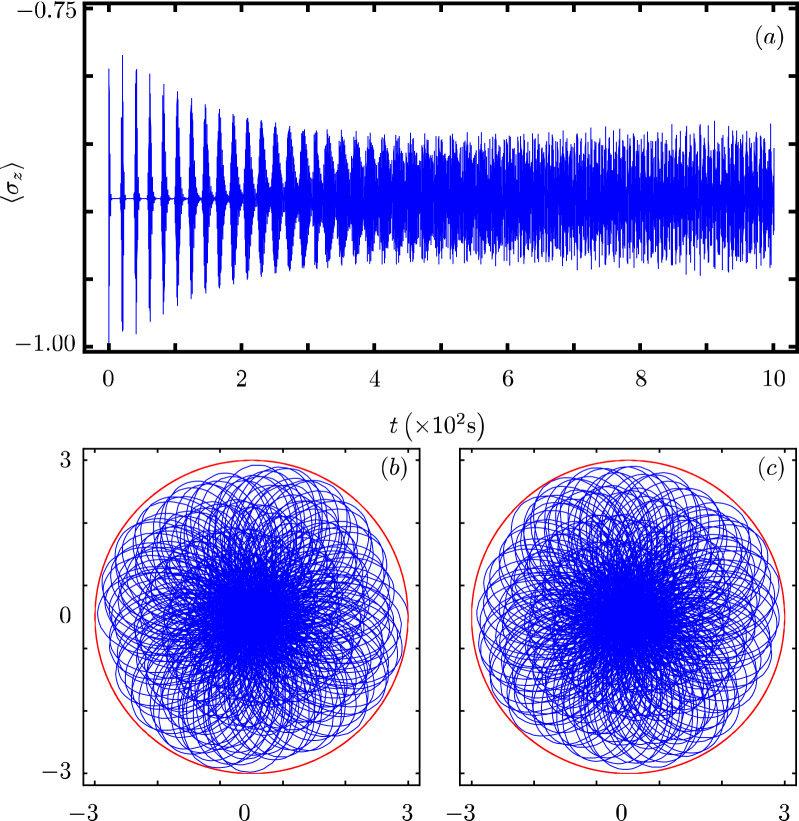
Same as Fig. 1 with parameters $G({\hat{n}})={\hat{n}}\frac{\beta _{2}+\beta _{1}}{2}$, $F({\hat{n}})={\hat{n}}\frac{\beta _{2}-\beta _{1}}{2}$, $k=2$, $\beta _{1}= \omega _{0}$, $\beta _{2}=0.75 \omega _{0}$.

The parameters $\alpha$ and $\beta$ control the new features and we identify $G({\hat{n}})={\hat{n}}\frac{\beta _2+\beta _{1}}{2}$, $F({\hat{n}})={\hat{n}}\frac{\beta _{2}-\beta _{1}}{2}$, $f({\hat{n}})=1$ and $k=2$. Its population inversion oscillates with a fast frequency and is highly localized around a value of zero, Fig. 3a. Its quadratures show that the boson state squeezes in a slower manner and explores more of the optical phase space than in the JC case, Fig. 3b,c.

### JC model with a Kerr medium

A single qubit in a single-mode cavity is surrounded by a Kerr-like medium^24,44–47^. The medium can be modeled as an anharmonic oscillator, the qubit undergoing two-photon transition is coupled to the cavity field which has a nonlinear interaction with the Kerr medium,

47 $$\begin{aligned} {\hat{H}}_{4}=\omega {\hat{n}}+\frac{\omega _0}{2}{\hat{\sigma }}_z+\chi {\hat{n}}({\hat{n}}-1) +g({\hat{\sigma }}_+{\hat{a}}^2+{\hat{\sigma }}_-{\hat{a}}^{\dagger 2}), \end{aligned}$$

where $\chi$ is a parameter controlling the strength of the Kerr term and we have the identification $G({\hat{n}})=\chi {\hat{n}}({\hat{n}}-1)$, $F({\hat{n}})=0$, $f({\hat{n}})=1$ and $k=2$.

**Figure 4:**
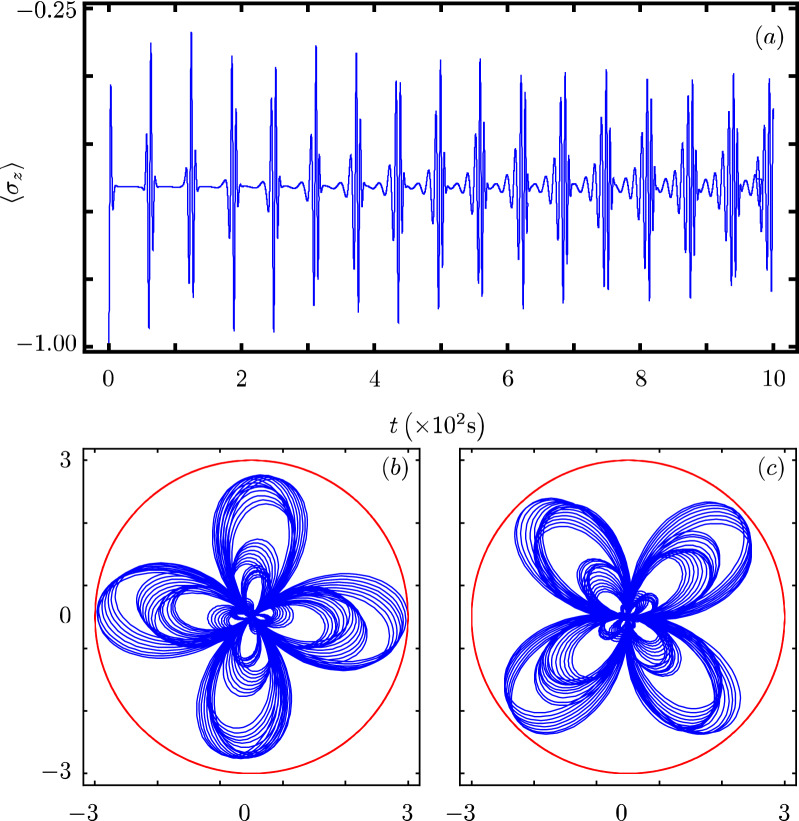
Same as Fig. 1 with $G({\hat{n}})=\chi {\hat{n}}({\hat{n}}-1)$, $k=2$, and $\chi =0.5 \omega _{0}$.

Its population inversion shows that the qubit state has periodical oscillations that bring it close to the initial state for small times, Fig. 4a. Its quadratures show that the boson state also approaches its original state, Fig. 4b,c.

### Molecular JC Hamiltonian

This model arises from molecular physics or from the nonlinear Jahn–Teller effect, although long-time behavior in either case might be obscured by omnipresent damping^25^,

48 $$\begin{aligned} {\hat{H}}_{5}=\omega {\hat{n}}+\frac{\omega _0}{2}{\hat{\sigma }}_z +\beta {\hat{n}}^2+g({\hat{\sigma }}_+{\hat{a}}+{\hat{\sigma }}_-{\hat{a}}^{\dagger }). \end{aligned}$$

The corresponding parameters are $G({\hat{n}})=\beta {\hat{n}}^2$, $F({\hat{n}})=0$, $f({\hat{n}})=1$ and $k=1$.

**Figure 5:**
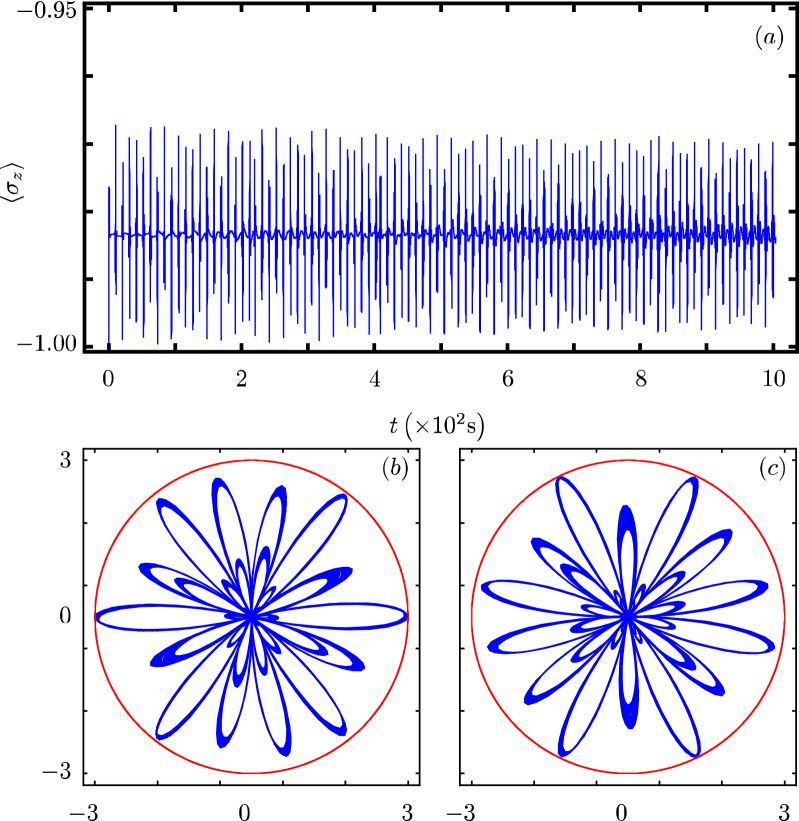
Same as Fig. 1 with $G({\hat{n}})=\beta {\hat{n}}^2$ and $\beta =0.3 \omega _{0}$.

Its population inversion shows that there is almost no energy exchange between the qubit and the boson, Fig. 5a. Its quadratures show that the boson state is squeezed and explores what seems a reduced portion of optical phase space, Fig. 5b,c.

### Algebraic JC model

**Figure 6:**
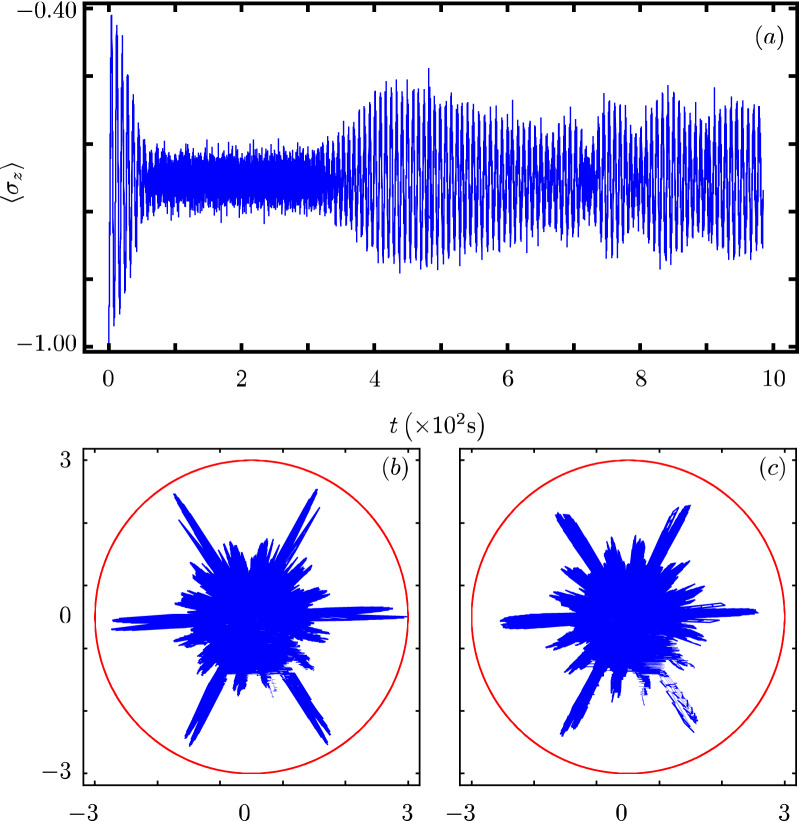
Same as Fig. 1 with $G({\hat{n}})=\chi _a {\hat{n}}({\hat{n}}^{\ell -1}-1)$, $f({\hat{n}})= \sqrt{1-\frac{\chi _a}{\omega }(1-{\hat{n}}^{\ell -1})}$, $\chi _{a}=0.5 \omega _{0}$, and $l=2$.

Here, a deformation of the boson mode operators introduces nonlinear exchange and nonlinear boson terms^48^,

49 $$\begin{aligned} {\hat{H}}_{6}=\omega {\hat{n}}+\frac{\omega _0}{2}{\hat{\sigma }}_z+\chi _a{\hat{n}}({\hat{n}}^{\ell -1}-1)+g\left( {\hat{\sigma }}_+{\hat{a}}\sqrt{1-\frac{\chi _a}{\omega }(1-{\hat{n}}^{\ell -1})}+{\hat{\sigma }}_-\sqrt{1-\frac{\chi _a}{\omega }(1-{\hat{n}}^{\ell -1})}{\hat{a}}^{\dagger }\right) , \end{aligned}$$

where the anharmonicity parameter fulfills $0\le \chi _a \ll \omega$ and $l\ge 1$. Here, we identify $G({\hat{n}})=\chi _a{\hat{n}}({\hat{n}}^{\ell -1}-1)$, $F({\hat{n}})=0$, $f({\hat{n}})=\sqrt{1-\frac{\chi _a}{\omega }(1-{\hat{n}}^{\ell -1})}$ and $k=1$. The population inversion presents localized oscillations around a negative value with high oscillation frequency, Fig. 6a. Its quadratures shows boson squeezing that is faster and more localized than in the standard JC model, Fig. 6b,c.

### Parity deformed JC model

**Figure 7:**
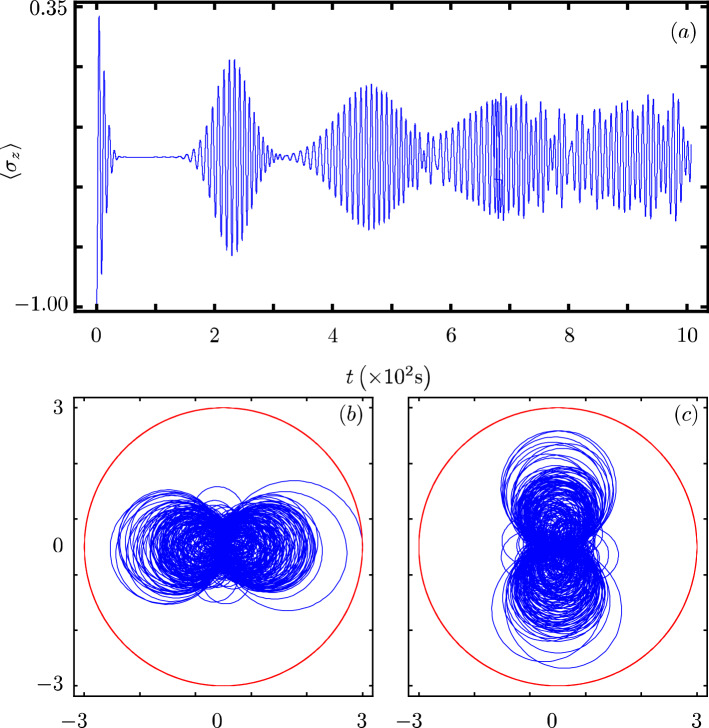
Same as Fig. 1 with $G({\hat{n}})= \lambda \left( -1 \right) ^{{\hat{n}}}$ and $\lambda =0.2 \omega _{0}$.

The parity deformed JC arises from a $\lambda$-analog of the Heisenberg algebra^49^,

50 $$\begin{aligned} {\hat{H}}_{7}=\omega {\hat{n}}+\frac{\omega _0}{2}{\hat{\sigma }}_z+\omega \lambda \left( -1\right) ^{{\hat{n}}} +g({\hat{\sigma }}_+{\hat{a}}+{\hat{\sigma }}_-{\hat{a}}^{\dagger }), \end{aligned}$$

where $\lambda$ is the deformation parameter and $\left( -1\right) ^{{\hat{n}}}$ is the parity operator. The functions defining the model are $G({\hat{n}})=\lambda \left( -1\right) ^{{\hat{n}}}$, $F({\hat{n}})=0$, $f({\hat{n}})=1$ and $k=1$. This is a curious model as its population inversion is similar to the JC model showing a collapse and revival but localized around a negative constant bias, Fig. 7a. Its quadratures shows boson squeezing that is faster and more localized than in the standard JC model but follow a similar evolution, Fig. 7b,c.

### q-Deformed JC model

**Figure 8:**
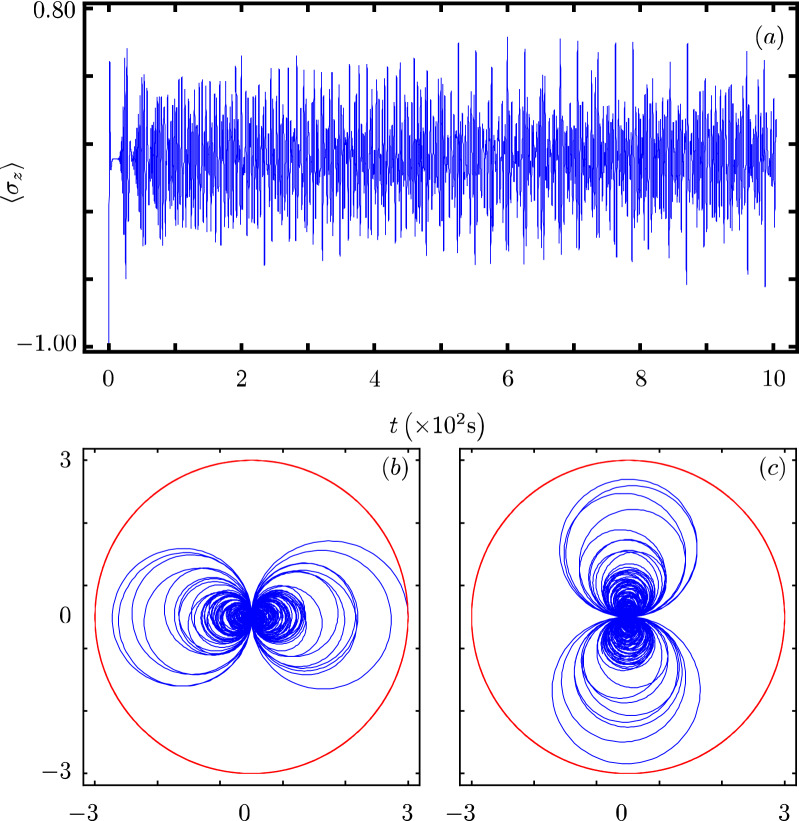
Same as Fig. 1 with $f({\hat{n}})=\sqrt{[{\hat{n}}]}$ and $q=0.9$.

This model implements deformed commutation relations for the boson operators that interpolates between Bose–Einstein and Fermi–Dirac commutation relations^50,51^,

51 $$\begin{aligned} {\hat{H}}_{8}=\omega {\hat{n}}+\omega _{0}{\hat{\sigma }}_{z}+g\left( \sigma _{-}\sqrt{[{\hat{n}}]}a^{\dagger }+\sigma _{+}a\sqrt{[{\hat{n}}]}\right) , \end{aligned}$$

where the deformed operator $[{\hat{n}}]$ is defined as

52 $$\begin{aligned}{}[{\hat{n}}]=\dfrac{q^{{\hat{n}}}-q^{-{\hat{n}}}}{q-q^{-1}}, \end{aligned}$$

in terms of the deformation parameter $q \le 1$. The corresponding parameters are $G({\hat{n}}) = 0$, $F({\hat{n}})=0$, $f({\hat{n}})=\sqrt{[{\hat{n}}]}$ and $k=1$. Its population inversion shows a high frequency oscillation without collapse nor revival, Fig. 8a. Its quadratures shows boson squeezing that is similar to that in the standard JC model but goes faster to a reduced optical phase space region, Fig. 8b,c.

## Conclusion

We started from the well-known analogy between supersymmetric quantum mechanics and the Jaynes–Cummings model to propose an extension that includes nonlinear boson processes, nonlinear dispersive interaction, and nonlinear multiboson exchange between the qubit and the boson. Our model helps realizing that the original Jaynes–Cummings model and most of its proposed extensions belong to a single Hamiltonian class.

We demonstrated that our model shows an underlying symmetry provided by a graded Lie algebra that has a similar behaviour to standard SUSY-QM. This structure allows us to construct a unitary transformation to diagonalize and provide analytic closed form eigenstates and eigenvalues as well as time evolution.

For the sake of providing a practical example, we used our closed form analytic expressions to explore the dynamics of models from the literature for an initial state where the qubit is in the ground state and the boson in a coherent state. While a detailed analysis is not within our scope, this allowed us to identify interesting dynamics in the population inversion and in the squeezing of the boson state. Some of these dynamics were unavailable at the time this manuscript was written.

As a final remark, we want to address the fact that it may seem cumbersome to use Lie’s program in a model that is feasible of diagonalization using standard techniques. We want to stress that discovering an underlying algebra may open the door to further analysis, for example the construction of ladder operators and their coherent states, or as a stepping stone to study more complex models, for example to explore transitions between particular models of the class that may be available using current quantum technologies.
